# LuxR Solos from Environmental Fluorescent Pseudomonads

**DOI:** 10.1128/mSphere.01322-20

**Published:** 2021-03-31

**Authors:** Cristina Bez, Sonia Covaceuszach, Iris Bertani, Kumari Sonal Choudhary, Vittorio Venturi

**Affiliations:** a International Centre for Genetic Engineering and Biotechnology, Trieste, Italy; b Institute of Crystallography, CNR, Trieste, Italy; c University of California San Diego, La Jolla, California, USA; University of Iowa

**Keywords:** LuxR solos, fluorescent *Pseudomonas*, quorum sensing

## Abstract

LuxR solos are related to quorum sensing (QS) LuxR family regulators; however, they lack a cognate LuxI family protein. LuxR solos are widespread and almost exclusively found in proteobacteria. In this study, we investigated the distribution and conservation of LuxR solos in the fluorescent pseudomonads group. Our analysis of more than 600 genomes revealed that the majority of fluorescent *Pseudomonas* spp. carry one or more LuxR solos, occurring considerably more frequently than complete LuxI/LuxR archetypical QS systems. Based on the adjacent gene context and conservation of the primary structure, nine subgroups of LuxR solos have been identified that are likely to be involved in the establishment of communication networks. Modeling analysis revealed that the majority of subgroups shows some substitutions at the invariant amino acids of the ligand-binding pocket of QS LuxRs, raising the possibility of binding to non-acyl-homoserine lactone (AHL) ligands. Several mutants and gene expression studies on some LuxR solos belonging to different subgroups were performed in order to shed light on their response. The commonality of LuxR solos among fluorescent pseudomonads is an indication of their important role in cell-cell signaling.

**IMPORTANCE** Cell-cell communication in bacteria is being extensively studied in simple settings and uses chemical signals and cognate regulators/receptors. Many Gram-negative proteobacteria use acyl-homoserine lactones (AHLs) synthesized by LuxI family proteins and cognate LuxR-type receptors to regulate their quorum sensing (QS) target loci. AHL-QS circuits are the best studied QS systems; however, many proteobacterial genomes also contain one or more LuxR solos, which are QS-related LuxR proteins which are unpaired to a cognate LuxI. A few LuxR solos have been implicated in intraspecies, interspecies, and interkingdom signaling. Here, we report that LuxR solo homologs occur considerably more frequently than complete LuxI/LuxR QS systems within the Pseudomonas fluorescens group of species and that they are characterized by different genomic organizations and primary structures and can be subdivided into several subgroups. The P. fluorescens group consists of more than 50 species, many of which are found in plant-associated environments. The role of LuxR solos in cell-cell signaling in fluorescent pseudomonads is discussed.

## INTRODUCTION

The recent development of omics methodologies and the extensive studies in microbial diversity have evidenced that, in nature, microbes live as part of complex mixed communities. For this reason, microbes very likely communicate and socially interact with numerous different species in order to cooperate, synchronize, and synergize their behavior in response to environmental changes. Quorum sensing (QS) is one type of social interaction among bacteria, which regulates gene expression in response to cell density, playing a major role in the formation and stability of microbial populations (1, 2). QS cell-cell signaling in bacteria has so far been mostly addressed in simple settings, mainly focusing on single species and thus limiting our understanding of its possible roles in complex mixed communities.

To date, the most common and best-studied QS system in Gram-negative proteobacteria is mediated by *N*-acyl homoserine lactone (AHL) signals. The archetypical AHL-QS system is composed by two most commonly genetically adjacent genes: the *luxI* family gene encoding an AHL synthase and its cognate *luxR* family gene, which encodes a transcriptional factor that detects and responds to the cognate AHL (3–5). LuxR-type family proteins are approximately 250 amino acids long and consist of two domains: an inducer-binding domain (IBD) at the N terminus (6, 7) and a DNA-binding helix-turn-helix (HTH) domain at the C terminus (8). The IBD of canonical LuxRs recognizes AHLs, resulting in a conformational change that affects its ability to bind target DNA in gene promoter regions at conserved sites, called *lux* boxes (9, 10). LuxRs share 9 highly conserved amino acid residues (11, 12). Six are hydrophobic or aromatic and form the cavity of the IBD, and the remaining three are in the HTH domain (12). LuxR family proteins are a source of adaptability and flexibility in QS circuits, allowing for alterations in response to AHL types or different signal molecules. In particular, signal specificity can be altered by specific changes in some residues of LuxR receptors (13). LuxRs can also be promiscuous by binding not only to their cognate AHL but also to multiple AHL types and thus responding to nonself signals (14). This eavesdropping through promiscuous receptors may play a role in interspecies interactions and can affect both interspecies competition and cooperation, expanding the function of QS systems in complex bacterial communities (15, 16).

Analysis of different genomes of proteobacteria has uncovered the widespread presence of LuxR regulators that occur without the cognate LuxI homolog; these are referred to as LuxR solos or orphans LuxRs (17–20). LuxR solos are closely related to QS LuxRs, displaying significant primary structure homology and having the same two-domain organization and modular structure as canonical LuxR proteins. LuxR solos can expand the regulatory targets by responding to endogenous or exogenous AHLs, also resulting in interspecies signaling. For example, QscR from Pseudomonas aeruginosa responds to endogenously produced AHLs (21, 22), SdiA of Salmonella enterica and Escherichia coli, which do not produce AHLs, responds to AHLs synthetized by neighboring bacteria (23–25), whereas PpoR from Pseudomonas putida binds to AHLs, either from self or foreign (26, 27).

LuxR solos have also been implicated in interkingdom signaling, having evolved to respond to signals produced by eukaryotes (28, 29). A subgroup of LuxR solos which is only found in plant-associated bacteria (PAB) responds to plant low-molecular-weight molecules (29–31). Compared to canonical QS LuxRs, members of this subfamily have some substitutions among the highly conserved amino acids in the IBD, which very likely correspond with their ability to bind low-molecular-weight compounds produced by plants (32). Members of this subfamily are found in both plant-pathogenic bacteria, such as XccR of Xanthomonas campestris, OryR of Xanthomonas oryzae, and XagR of Xanthomonas axonopodis, and beneficial bacteria, such as NesR in Sinorhizobium meliloti, PsoR of rhizospheric Pseudomonas fluorescens, PipR of plant-endophytic *Pseudomonas* sp. strain GM79, and PsrR of *Kosakonia* sp. strain KO348 (19, 26, 32–36). Finally, the LuxR solos PluR and PauR from the insect pathogen Photorhabdus luminescens and human and insect pathogen Photorhabdus asymbiotica, respectively, respond to novel endogenous molecules, namely, photopyrones and dialkylresorcinols (37–39). In summary, LuxR solos extend beyond next-of-kin AHL-driven QS, being used in different ways by bacteria and thus becoming major players in cell-cell communication in bacteria (37, 40).

In this study, we intend to begin to map LuxR solos in the model proteobacterial *Pseudomonas* genus by genomics and genetics/molecular biology approaches. The distribution, conservation, and possible responses of a set of LuxR solos within the P. fluorescens group were investigated. This group of bacteria is one of the most diverse groups within the *Pseudomonas* genus, comprising more than 50 species and many unclassified isolates, many of which are found in plant-associated environments (41, 42). Many members of the fluorescent *Pseudomonas* spp. are excellent rhizosphere colonizers and are studied for their plant-beneficial properties (43). An analysis for QS LuxRs domains of more than 600 genomes has evidenced the predominance of LuxR solos in fluorescent pseudomonads, which were divided into nine different subgroups based on their neighboring genes and their primary structure. The cartography of their ligand-binding sites allowed us to classify each LuxR solo into potential AHL-binding or non-AHL-binding types. LuxR solo genomic knockout mutants in several *Pseudomonas* sp. strains of different subgroups have been generated and studied in order to get insights into possible gene targets and mechanisms of action. Overall, our analysis revealed that LuxR solo homologs occur considerably more frequently than complete LuxI/LuxR QS systems within the P. fluorescens group and that LuxR solos from closely related genomes or from genomes carrying multiple LuxR solos cluster in different subgroups. These results highlight the existence of novel and diverse LuxR solo subgroups, which could be involved in intercellular (cell-cell) or intracellular signaling regulatory functions. Some could have evolved away from canonical QS LuxRs and possibly bind to new signals/molecules.

## RESULTS

### QS LuxRs and LuxR solos in the genomes of environmental fluorescent pseudomonads.

To investigate the presence, distribution, and conservation of QS LuxRs among the P. fluorescens complex, a systematic bioinformatic analysis has been performed. A collection of 601 sequenced genomes belonging to 17 different fluorescent pseudomonad species were sourced from the PATRIC database (44) and analyzed to identify putative LuxR solos, according to the criteria described in Materials and Methods. All potential QS LuxRs and LuxR solos identified contained the typical two signature Pfam domains: PF03472 autoind_bind domain at the N terminus and PF00196 DNA-binding HTH domain at the C terminus. However, the nine signature conserved residues (six key amino acids in the inducer-binding domain and three key amino acids in DNA-binding domain) found in archetypical QS LuxRs were not all present in many of the LuxR solos detected.

In total, 651 QS LuxR protein sequence hits were identified consisting of 528 LuxR solos and 123 LuxR proteins that are part of 122 complete QS systems (one system had a LuxR-LuxI-LuxR configuration). Of 601 fluorescent *Pseudomonas* genomes analyzed, only 87 genomes (14.5%) contained complete QS LuxI/R systems (a few genomes had multiple complete QS systems). On the other hand, more than half (approximately 50.5%; 303 genomes) harbor at least one LuxR solo, while only 8.9% of the genomes (55 genomes of 601 total) contain both LuxR solos and a complete QS system(s) (see Table S2 in the supplemental material). In approximately 35% of genomes, we did not detect either a complete QS LuxI/R system or a LuxR solo.

10.1128/mSphere.01322-20.6 TABLE S2 Complete table describing details for each LuxR solo protein detected among the 601 fluorescent *Pseudomonas* genomes. Download Table S2, XLSX file, 0.06 MB. Copyright © 2021 Bez et al. 2021 Bez et al. https://creativecommons.org/licenses/by/4.0/ This content is distributed under the terms of the Creative Commons Attribution 4.0 International license.

The vast majority of fluorescent *Pseudomonas* genomes carried more than one copy of a QS *luxR* solo gene. In this regard, the most varied distribution was found in strains belonging to P. fluorescens and P. putida, which could carry up to four *luxR* solo genes (Table S2). Overall, these observations show that it is much more common for fluorescent pseudomonads to harbor LuxR solo proteins than complete QS system(s).

It was also of interest to further analyze the conservation and distribution of the QS LuxR solos among the fluorescent pseudomonads isolated from plant roots. For this purpose, several fluorescent pseudomonad strains have been isolated from the rhizosphere of rice plants, as described in Materials and Methods. The complete genomes of 20 strains have been determined and mined for QS LuxR solos. None of the genomes carried a complete QS *luxI/R* system(s), whereas all harbored one or multiple *luxR* solos (see Table S3). This observation suggested a clear trend for the occurrence of LuxR solos, which could play a role in adaptation in the plant root habitat. In summary, this analysis of 621 fluorescent pseudomonads (601 genomes downloaded from PATRIC and 20 genomes sequenced in this study) highlights that QS LuxI/R systems are not abundantly present. In contrast, LuxR solos are prevalent, indicating a probable evolution away from complete AHL QS LuxI/R systems.

10.1128/mSphere.01322-20.7 TABLE S3 Complete table describing details for each LuxR solo protein detected among the 20 rhizospheric fluorescent *Pseudomonas* genomes. Download Table S3, XLSX file, 0.01 MB. Copyright © 2021 Bez et al. 2021 Bez et al. https://creativecommons.org/licenses/by/4.0/ This content is distributed under the terms of the Creative Commons Attribution 4.0 International license.

### Phylogenetic analysis and functional grouping of LuxR solos of the environmental fluorescent pseudomonads.

To determine the relatedness between the LuxR solos identified in fluorescent pseudomonads, a phylogenetic analysis was carried out, as detailed in Materials and Methods. Several clades clearly emerged based on their primary structure, as evidenced by the phylogenetic tree (see Fig. S1). Interestingly, these LuxR solo clades do not cluster according to the species taxonomy, since several branches of the tree are formed by LuxR solos belonging to different fluorescent pseudomonad species. In addition, multiple LuxR solos carried by the same genome do not cluster together, indicating low relatedness.

10.1128/mSphere.01322-20.1 FIG S1 Phylogenetic analyses of multiple LuxR solos as described in Results. The tree was inferred by using the maximum likelihood method. Colors indicate bacterial species. Download FIG S1, PDF file, 0.09 MB. Copyright © 2021 Bez et al. 2021 Bez et al. https://creativecommons.org/licenses/by/4.0/ This content is distributed under the terms of the Creative Commons Attribution 4.0 International license.

**FIG 1:**
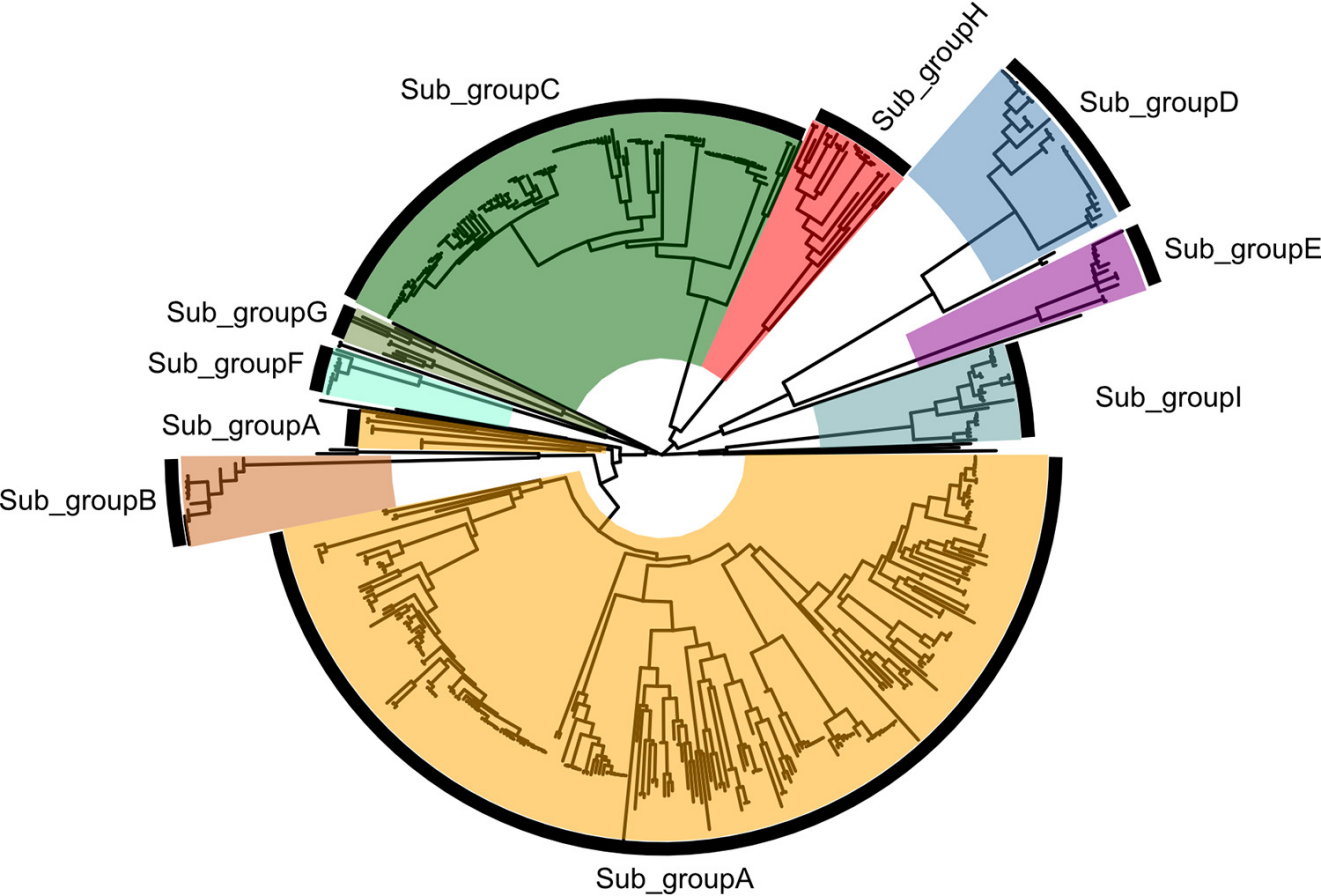
Phylogenetic analysis and functional grouping of 528 LuxR solos carried by fluorescent *Pseudomonas.* Subgroups are highlighted with a different colored background. LuxR solos which did not fit in any of the subgroups are not labeled.

**FIG 2:**
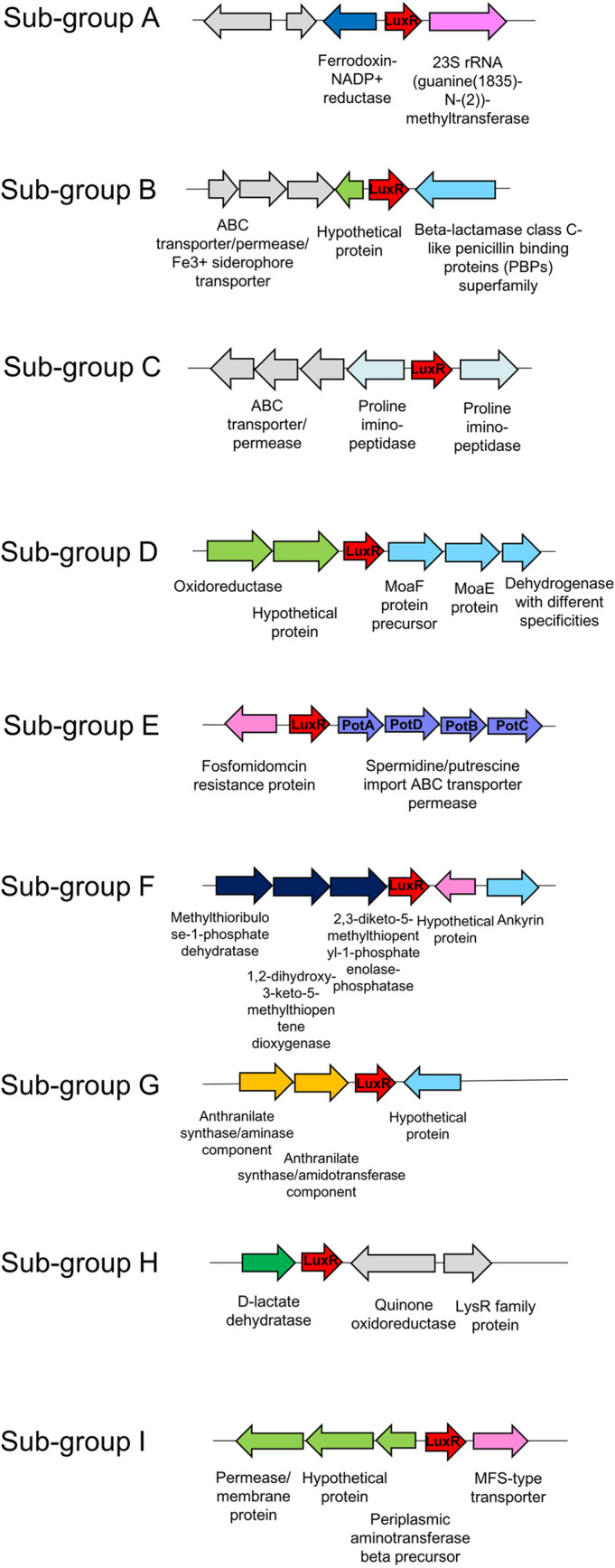
Functional grouping of LuxR solos and genomic context (5 kb).

It was also of interest to classify closely related LuxR solos into putative functional groups in order to further understand their relatedness and gain insights on their biological role. For this reason, the analysis of the genomic context flanking each LuxR solo was performed, since in bacteria, it is common that adjacent loci are targets for the transcriptional regulators. The primary structure and adjacent loci allowed LuxR solos to be divided into nine different subgroups (Fig. 1). Comembers of the subgroups are likely orthologs and functionally related, and almost all identified putative LuxR solos could be placed into these nine subgroups (Fig. 2). Only a few remained ungrouped, showing unique primary structure and flanking gene context (see Fig. S2). LuxR solos were (i) highly conserved within the subgroups B and F (sequence homology between 99.5% and 100%), (ii) medium conserved within the subgroups D, E, H, and I (75% to 90%), and (iii) low conserved within subgroups A and G (31% to 52%). LuxR solos belonging to different subgroups showed a sequence relatedness of around 10% to 25% (see Table S4). As previously mentioned, LuxR solos belonging to the same subgroup were found in different taxonomic clades of the phylogenetic tree; the nine LuxR solo subgroups are discussed below.

10.1128/mSphere.01322-20.2 FIG S2 The same phylogenetic analysis presented in Fig. S1. In this phylogenetic tree, each LuxR solo hit is named with the full ID and highlighted with a different color according to the subgroup they belong to, as described in Results. LuxR solos which did not fit in any of the subgroups are labeled in black. Download FIG S2, PDF file, 0.09 MB. Copyright © 2021 Bez et al. 2021 Bez et al. https://creativecommons.org/licenses/by/4.0/ This content is distributed under the terms of the Creative Commons Attribution 4.0 International license.

10.1128/mSphere.01322-20.8 TABLE S4 Overview of the primary structure homologies among the different subgroups of LuxR solos using the pairwise comparison. Download Table S4, PDF file, 0.1 MB. Copyright © 2021 Bez et al. 2021 Bez et al. https://creativecommons.org/licenses/by/4.0/ This content is distributed under the terms of the Creative Commons Attribution 4.0 International license.

### (i) Subgroup A.

LuxR solos of this subgroup occur in almost all the fluorescent *Pseudomonas* species analyzed here. Two very conserved genes always flank these LuxR solos, (i) encoding a ferredoxin-NADP^+^ reductase and (ii) encoding a 23S rRNA methyltransferase; for this reason, it is likely that these adjacent loci are functionally associated with the flanking *luxR* solo (Fig. 2). Either the ferredoxin-NADP^+^ reductase or the 23S rRNA methyltransferase is involved in primary metabolism participating in a wide variety of redox metabolic pathways, suggesting a possible role for the LuxR solo in regulating a broad range of key metabolic functions. This LuxR solo and the adjacent loci are also highly conserved in all the 20 rice rhizosphere genomes isolated and sequenced in this study (Fig. S2; Table S3).

### (ii) Subgroup B.

The subgroup B is only found in Pseudomonas protegens species. The neighboring genes are beta-lactamase class C-like protein on one side and a hypothetical protein of unknown function on the other side (Fig. 2; Fig. S2).

### (iii) Subgroup C.

This subgroup of LuxR solos is well studied and is often referred to as PAB LuxR solos that respond to plant low-molecular-weight compounds. They are found in many different species of plant-associated bacteria (35); examples are OryR and XccR, which are found in *Xanthomonas* plant pathogens, and PipR and PpoR, which are harbored in plant-beneficial *Pseudomonas* sp. (26, 32–34). These LuxR solos show some substitutions among the highly conserved amino acid residues in the IBD binding pocket and regulate the adjacently located proline iminopeptidase (*pip*) gene. By responding to plant compound(s), these LuxR solos constitute an interkingdom signaling circuit involved in plant-bacteria interactions (31).

### (iv) Subgroup D.

This is a small subgroup, which is not frequent among *Pseudomonas* species. These LuxR solos are flanked by two operons with hypothetical functions, most probably involved in primary metabolism. One operon consists of an oxidoreductase and a hypothetical protein and the other encodes Moa-like proteins, which are likely to be involved in the biosynthesis of the molybdopterin cofactor (MoCo) that is fundamental for the activity of many important enzymes processes (45) (Fig. 2).

### (v) Subgroup E.

These LuxR solos are harbored by several different fluorescent pseudomonad species (Fig. S2) and are flanked by genes involved in polyamine membrane transport. Polyamines are aliphatic polycationic molecules (i.e., spermidine, spermine, and putrescine), which are widely distributed in bacteria, plants, and animals and have been implicated as signaling molecules not only between microorganisms but also in the interkingdom cell-cell communication (46–48). This group of LuxR solos might therefore be involved in the response to polyamine molecules and possibly in plant-bacteria communication.

### (vi) Subgroup F.

This subgroup was limited to the Pseudomonas viridiflava species, possibly suggesting a very specific function for this LuxR solo in regulating currently unknown mechanisms for its lifestyle. The adjacent loci consist of an upstream operon of three genes involved in the l-methionine biosynthesis pathway and a downstream gene coding for an ankyrin-type protein (Fig. 2; Fig. S1 and S2).

### (vii) Subgroup G.

This subgroup is characterized by the presence of an adjacent operon of two genes encoding the anthranilate synthase enzymes, which are involved in phenylalanine/tyrosine metabolism (Fig. 2). These enzymes catalyze the conversion of chorismate into anthranilate, the biosynthetic precursor of tryptophan and numerous other secondary metabolites. Thus, it is a possibility that the operon flanking this LuxR solo might be involved in the synthesis of signal molecules.

### (viii) Subgroup H.

This subfamily consists of the *luxR* solo as part of an operon with a d-lactate dehydrogenase gene that encodes an enzyme which belongs to the oxidoreductase family and participates in pyruvate metabolism. This subgroup has been found in a small number of *Pseudomonas* species (Fig. 2; Fig. S2).

### (ix) Subgroup I.

This subgroup is formed by LuxR solos that are located adjacent to two different loci, upstream and downstream, that both encode transporter or permease proteins (Fig. 2). It is therefore possible that these LuxR solos regulate loci that affect the movement of compounds/molecules through the bacterial membrane.

In summary, these observations revealed that LuxR solos are predominant in fluorescent pseudomonads and allowed classification into several subgroups based on the conservation in their primary structures and neighboring loci.

### Comparative cartographic analysis of the identified subgroups of LuxR solos in fluorescent pseudomonads.

**FIG 3:**
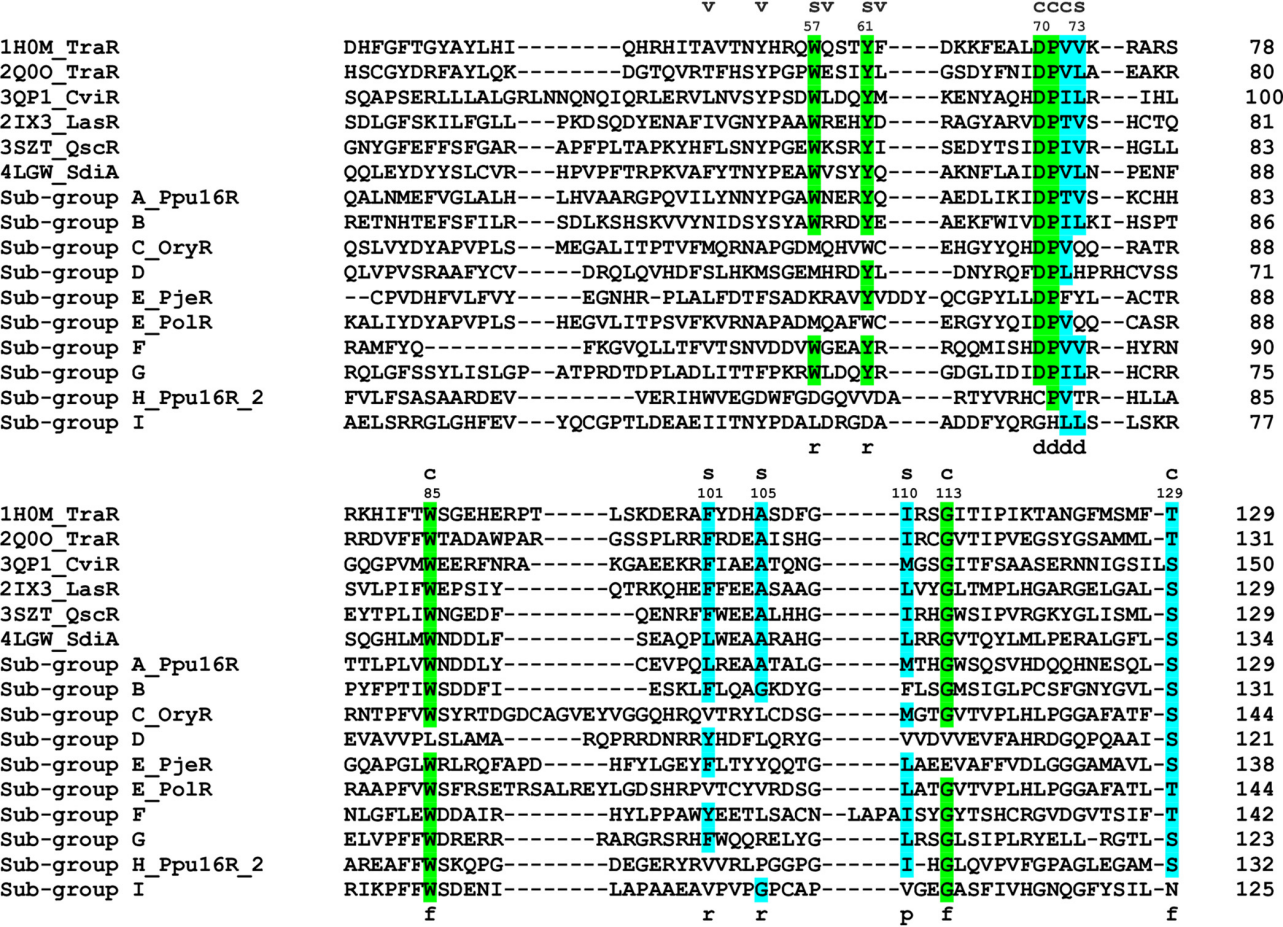
Structure-based multiple sequence alignment of the inducer-binding domains of the prototypes of the nine identified LuxR solo subgroups with QS LuxRs. The residues belonging to cluster 1 and cluster 2 are highlighted in green and cyan, respectively. The 3D architecture of the boundaries of the ligand-binding site is schematized by r (roof), f (floor), p (proximal wall), and d (distal wall) and its tripartite topology by c (conserved core), s (specificity patch), and v (variable patch).

To gain insights into the architecture of the LuxR solo inducer-binding pockets and their signal specificity, we have applied a cartographic analysis of the selected solos based on structure-based homology modeling and structural superimposition, combined with multiple structure-based sequence alignments. Previous studies have found that signal specificity could be altered by specific substitutions of conserved amino acids within the inducer-binding domain (IBD). In particular, we focused on the pocket residues directly interacting with the ligand that are conserved and belong to cluster 1 and cluster 2 (colored in green and in cyan, respectively, in Fig. 3), as previously described (49). Residues of the third cluster, belonging to a variable patch and thus being poorly conserved even within members of QS LuxRs, have not been taken into account.

**FIG 4:**
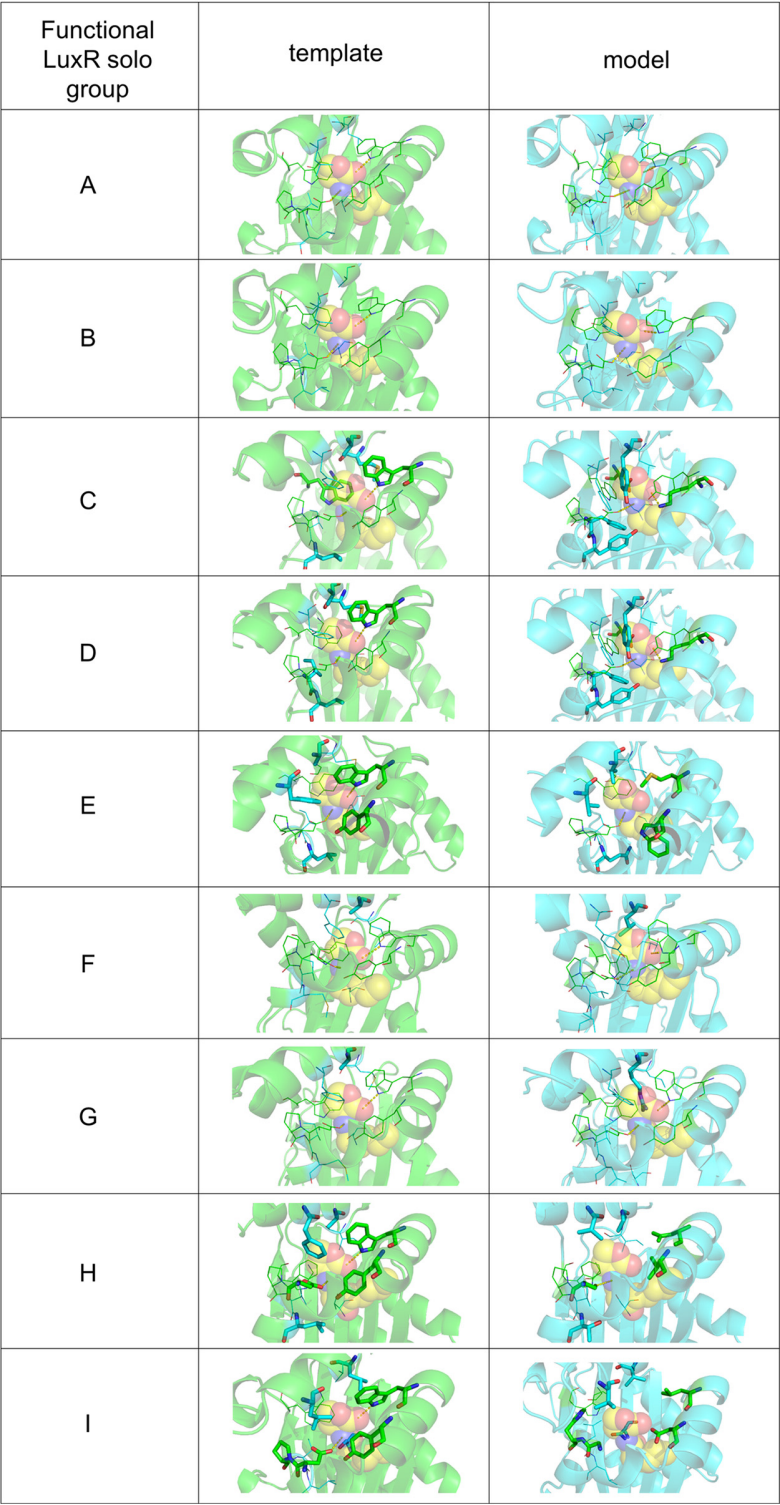
Comparison of the inducer-binding sites of the prototypes of the nine identified LuxR solo subgroups (right column) with the corresponding QS LuxRs templates used for their *in silico* modeling (left column). Semitransparent cartoon representation, with the side chains of residues belonging to cluster 1 and cluster 2 highlighted in green and cyan, respectively: conserved residues are represented by lines, while nonconserved amino acids are highlighted by sticks. The bound AHL is represented by spheres and its carbon, nitrogen, and oxygen atoms are colored in yellow, blue, and red, respectively. The hydrogen bonds stabilizing the lactone ring binding are highlighted by yellow dotted lines. Figures produced by PyMOL (version 1.3 r1; Schrödinger LLC).

We have selected 10 LuxR solos that represent each of the nine subgroups discussed above and have analyzed the molecular determinants of each inducer-binding site. This analysis revealed key differences between the binding sites among the representatives of each subgroup (Fig. 3 and 4): all the comparisons were paralleled to TraR from Agrobacterium tumefaciens, as the prototype of QS LuxR proteins. According to the molecular cartography and structure-based alignment, only two subgroups (A and B) are very closely related to the archetypical QS LuxRs. They maintained the two conserved hydrogen bonds stabilizing AHL binding (Fig. 3 and 4), namely, one between the ε nitrogen of W57 (according to TraR numbering) and the carbonyl oxygen of the lactone moiety and the second between the ε oxygen of D70 and the nitrogen preceding the acyl moiety. In addition, all the apolar residues belonging to the conserved and specificity patches, which further stabilize the AHL binding by hydrophobic interactions, are maintained with respect to the AHL binding template, except for one substitution of a residue with similar steric hindrance (L→M). Overall, the cartographic observations suggest that these two subgroups of LuxR solos very likely bind and respond to AHLs.

Interestingly, the binding pockets of the members of subgroup F and G are characterized by an overall conservation in all the residues of the conserved and specificity patches, except for the amino acid corresponding to A105 of TraR. The substitutions of this small side chain with residues characterized by much higher steric hindrance (A→L and A→R in subgroups F and G, respectively) deeply impact the shape of the binding sites, partially occluding the hydrophobic pocket in which the lactone ring accommodates. This effect due to a single substitution is very likely to alter the ligand specificity of subgroups F and G with respect to canonical AHL-binding LuxRs.

The members of subgroup C, which has been already identified as a member of the PAB LuxR solo group that respond to plant compounds, as previously reported, showed replacement of amino acids at positions corresponding to the following residues of TraR: W57 (→M, leading to the loss of one of the stabilizing hydrogen bonds), V73 (→Q, impacting the hydrophobic environment of the cleft), Y61 (→W), F101 (→V), and A105 (→L), which generate different steric hindrances, likely altering the shape of the pocket. Overall, these key differences suggest a different specificity toward what is believed to be a plant compound(s) (30, 33).

Surprisingly, the remaining groups showed significant modifications in the binding pocket due to several changes not only in the specificity patch but also in the invariant amino acids of the conserved patch, thus suggesting that these proteins likely bind other non-AHL compounds (Fig. 4). In particular, all the candidates from the latter groups have lost at least one of the two hydrogen bonds stabilizing AHL binding, due to substitutions not only in the residue corresponding to W57 of TraR, which is part of the specificity patch and is not conserved in all 4 subgroups, but also in the very conserved amino acid corresponding to D70 of TraR, namely, in subgroups H and I. Additional invariant positions that are not conserved in these subgroups are the ones corresponding to TraR V72 (→F in PjerR of subgroup E, leading to increased steric hindrance), W85 (→L in subgroup D, leading to decreased steric hindrance), and G113 (→E in PjerR of subgroup E and →V in subgroup D, leading to increased steric hindrance that is also combined with huge variation in the electrostatics of the pocket in the case of PjeR). Regarding the specificity patch, the residues at almost all the positions are substituted with amino acids with side chains that have entirely different steric hindrance and, moreover, are charged or polar, profoundly impacting not only the overall shape but also the hydrophobicity of the pocket that is a prerequisite for AHL binding (Fig. 3). Therefore, these subgroups of LuxR solos appear to be more distantly related to the canonical QS LuxRs and are possibly able to respond to yet-unknown exogenous or endogenous compounds.

In summary, the cartographic analysis showed variable degrees of conservation in the amino acids forming the binding pocket among the LuxR solos of the fluorescent pseudomonads. Thus, we hypothesize that some eavesdrop by binding AHLs, whereas others could have evolved to bind different compounds/signals produced by neighboring species or possibly currently unknown endogenous signals.

### Potential target gene promoter expression analysis of a set of LuxR solos.

**FIG 5:**
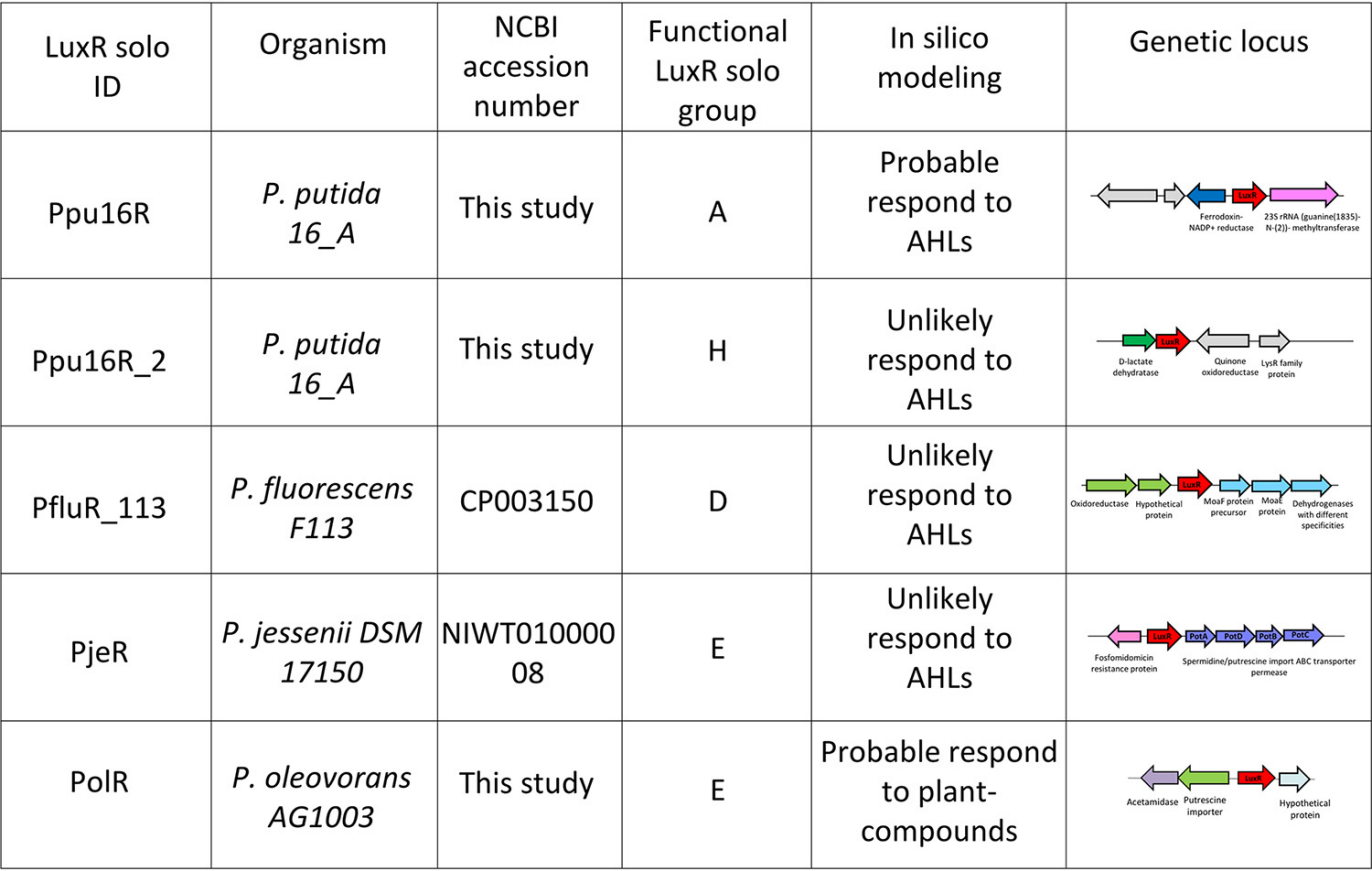
LuxR solos candidates for target gene promoter expression analyses.

To begin to acquire insights into the mode of action of the LuxR solos, *luxR* solo autoregulation and gene expression studies of the flanking loci were performed. LuxR solo candidates from subgroups A, D, E, and H were selected as they showed some interesting features according to our analyses (Fig. 5). The *luxR* solo genes were mutated in fluorescent pseudomonad strains, as described in Materials and Methods section and Table S1. The transcription of the various loci was studied via gene promoters transcriptionally fused to a *lacZ* reporter gene in a plasmid construct, and assays were performed in the wild-type and *luxR* solo mutant strains. Below, we present the results of these studies on five LuxR solos that belong to the four different subgroups.

10.1128/mSphere.01322-20.5 TABLE S1 Sequence design of each *luxR* solo gene mutant. Download Table S1, XLSX file, 0.01 MB. Copyright © 2021 Bez et al. 2021 Bez et al. https://creativecommons.org/licenses/by/4.0/ This content is distributed under the terms of the Creative Commons Attribution 4.0 International license.

### (i) Ppu16R of subgroup A.

Ppu16R of P. putida 16A is highly identical in its IBD to QS-LuxRs, and cartographic analysis predicted that it very likely binds and responds to AHLs. Therefore, it was of interest to study its autoexpression and that of the adjacent genes in the presence/absence of AHLs. Moreover, mining the genome of P. putida 16A revealed that it does not possess any canonical AHL-QS LuxI/R systems, suggesting Ppu16R could be responding to exogenous AHLs. No Ppu16R-dependent promoter activities were detected in either the presence or absence of AHLs under the conditions tested here (see Fig. S3a). Possible explanations are that the Ppu16R does not autoregulate its expression and that flanking genetic loci are not its targets or the conditions used in this study do not allow for activating/repressing the expression of these genes. To further investigate whether Ppu16R can bind AHLs, His-tagged Ppu16R was recombinantly expressed in E. coli in the presence of different AHLs, as most commonly, AHL-binding QS LuxRs are stabilized and solubilized when bound to AHLs (5). The His-tag resulted in Ppu16R being soluble in the absence of AHLs, and the presence of AHLs did not increase solubility (data not shown), not allowing a direct readout of AHL binding (Fig. S4). This LuxR protein solubility independent of AHLs was also observed for the SdiA LuxR from E. coli (50). In summary, these studies have not provided direct evidence for gene targets and AHL binding for this LuxR solo.

10.1128/mSphere.01322-20.3 FIG S3 (a) Gene promoter activity in the presence or absence of AHLs in P. putida 16A wild type (WT) and Δ*Ppu16R.* β-Galactosidase activities (Miller units) of 3-gene promoter transcriptional fusion (*ppu16R*, ferredoxin NADP^+^ reductase, and 23S methyltransferase) were determined to compare the expression levels between WT and Δ*Ppu16R* stains. The WT and Δ*Ppu16R* strain with empty plasmid pMP220 were used as controls. The AHLs used are the following: linear AHLs (C_4_-AHL, C_6_-AHL, C_8_-AHL, C_10_-AHL, and C_12_-AHL), OH-AHLs (OHC_6_-AHL, OHC_8_-AHL, and OHC_12_-AHL), and O-AHLs (OC_6_-AHL, OC_8_-AHL, andOC_12_-AHL). (b) Gene promoter activity in P. fluorescens F113 and Δ*pfluR* strains. β-Galactosidase activities (Miller units) of 2-gene promoter transcriptional fusion (p*fluR moaF*) were determined to compare the expression levels between WT and Δ*pfluR* strains. The WT and Δ*pfluR* strain with empty plasmid pMP220 were used as controls. The promoter activity was calculated after 4 h (log phase) and after overnight growth (stationary phase). (c) Gene promoter activity in the presence or absence of a cocktail of polyamines (putrescine, spermine, and spermidine) at a final concentration of 0.1 mM in *P. jessenii* DSM 17150 and the Δ*pjeR* strain. β-Galactosidase activities (Miller units) of 2-gene promoter transcriptional fusion (*pjeR* and spermidine transporter) were determined to compare the expression levels between WT and Δ*pjeR* strains. The WT and Δ*pjeR* strain with empty plasmid pMP220 were used as controls. (d) Gene promoter activity in the presence or absence of a cocktail of polyamines (putrescine, spermine, and spermidine) at a final concentration of 0.1 mM in *P. oleovorans* AG1003 and the Δ*polR* strain. β-Galactosidase activities (Miller units) of 2-gene promoter transcriptional fusion (*polR* and putrescine importer) were determined to compare the expression levels between WT and Δ*polR* strains. The WT and Δ*polR* strain with empty plasmid pMP220 were used as controls. (e) Gene promoter activity in P. putida 16A and Δ*ppuR_2* strains. β-Galactosidase activities (Miller units) of the *ppuR_2* promoter transcriptional fusion were determined to compare the expression levels between WT and Δ*ppuR_2* strains. The WT and Δ*ppuR_2* strain with empty plasmid pMP220 were used as controls. The promoter activity was calculated after 4 h (log phase) and after overnight growth (stationary phase). All experiments were performed in triplicates. Statistical analysis was calculated using one-way ANOVA followed by Tukey’s multiple-comparison test by Prism 7 (GraphPad Software, Inc.). The error bars indicate standard deviations. Download FIG S3, TIF file, 1.1 MB. Copyright © 2021 Bez et al. 2021 Bez et al. https://creativecommons.org/licenses/by/4.0/ This content is distributed under the terms of the Creative Commons Attribution 4.0 International license.

10.1128/mSphere.01322-20.4 FIG S4 His-tagged *Ppu16R* expression in pETM-11 adding 100 mM of each AHL (C_6_-AHL, C_8_-AHL, and C_14_-AHL). Soluble fractions purified using MagneHis protein purification system (Promega Corp., Madison, WI, USA) is shown in the protein gel; *Ppu16R* was soluble when unbound to AHLs. Download FIG S4, TIF file, 0.1 MB. Copyright © 2021 Bez et al. 2021 Bez et al. https://creativecommons.org/licenses/by/4.0/ This content is distributed under the terms of the Creative Commons Attribution 4.0 International license.

### (ii) PfluR113 of subgroup D.

The PfluR113 solo of P. fluorescens F113 belonged to subgroup D, and according to cartographic analysis, it most probably does not bind AHL signals. In addition, this strain does not possess any canonical AHL-QS LuxI/R systems. To understand whether there was autoregulation and whether adjacent operons were regulated by the nearby solo gene, we determined the transcriptional activity of *pfluR113* and the adjacent operons. This established that PfluR113 negatively regulated the transcription of one of the genetically linked operons. A significant increase of the expression of the operon in the Δ*pfluR_113* mutant was determined when the bacterial culture was in an early log phase, while no significant differences were detected in the stationary phase (Fig. S3b). Complementation of the Δ*pfluR_113*, via the wild-type gene harbored in a plasmid, restored the expression levels observed in the wild-type strain in the early log phase. This suggested that PfluR113 plays a role in the growth phase-dependent regulation of the adjacent operon and that this solo may respond to some yet-uncharacterized endogenous signals/molecules.

### (iii) PjeR and PolR of subgroup E.

It was of interest to investigate whether polyamines could bind to the LuxR solos belonging to subgroup E, since they were flanked by genes most likely involved in transporting polyamines through the bacterial membrane. Several recent studies have shown that polyamines (i.e., putrescine, spermidine, and spermine) play a role in cell-to-cell signaling regulating phenotypes such as surface motility, biofilm formation, and cell differentiation (51, 52). Moreover, according to the modeling of the ligand-binding pocket, this LuxR solo subgroup most likely responds to non-AHL molecules. As described in Fig. 5, we tested the expression of *pjeR* from Pseudomonas jessenii DSM 17150 and of the adjacent putative spermidine transporter gene. Similarly, we also tested the expression of *polR* from Pseudomonas oleovorans AG1003 and the flanking putative putrescine importer gene. *P. jessenii* DSM 17150 and *P. oleovorans* AG1003 do not possess any canonical AHL-QS LuxI/R systems. All the promoter activities were examined in the presence or absence of (i) putrescine, (ii) spermidine, and (iii) spermine. The results showed that none of these gene promoters were activated/induced under any conditions tested (Fig. S3c and S3d).

### (iv) Ppu16R2 of subgroup H.

Ppu16R2 is a second LuxR solo harbored by P. putida 16A that constitutes an operon with the d-lactate dehydrogenase gene. In this subgroup, the operonic structure is always conserved, suggesting a potential role for this LuxR in pyruvate metabolism via the glyoxalase pathway. The results obtained (Fig. S3e) showed no *ppu16R2* autoregulation of the operon in either the early log phase or stationary phase.

In summary, these studies revealed that, most commonly, the *luxR* solos are not autoregulated and do not regulate adjacent genes under the conditions that were tested here (Fig. S3d).

## DISCUSSION

QS LuxR solos are a subfamily of QS LuxR proteins that are very widespread in proteobacteria and maintain the N-terminal IBD and C-terminal HTH domains and occur without a cognate LuxI-AHL synthase. To date, only a few LuxR solos have been studied, which has shown that they can be involved in intraspecies, interspecies, and interkingdom signaling.

In this study, we investigated the distribution and conservation of LuxR solos among members of the fluorescent *Pseudomonas* group, many of which are plant commensals being studied for their biocontrol and plant growth promotion properties (53). Our analysis of more than 600 genomes revealed that the majority of fluorescent *Pseudomonas* spp. carry one or more LuxR solos. We have clustered them into nine subgroups based on their adjacent gene context and primary structure. The modeling analysis revealed that the majority show substitutions at the invariant amino acids of the ligand-binding pocket, raising the possibility of binding to non-AHL ligands or function independent of any ligand.

Only 14.5% of the fluorescent *Pseudomonas* spp. analyzed harbor a complete AHL QS system in their genomes, whereas more than half (50.5%) harbor only *luxR* solos. This result is in line with a previous study (20) that demonstrated that many *Gammaproteobacteria* carried multiple LuxR solos, particularly plant-associated and environmental isolates. In addition, our isolation and analysis of a set of 20 rice rhizospheric P. fluorescens isolates further confirmed the trend for the high occurrence of LuxR solos, since we have identified only *luxR* solo genes among these genomes and no complete AHL QS systems. This result suggests a specific role for single or multiple LuxR solos in bacterial species that colonize plant-associated niches. Rhizosphere *Pseudomonas* spp. rarely harbor a complete AHL QS system, and its lack of conservation and the unpredictable role played indicates that it is not part of the core genome (54). The absence of complete canonical LuxI/LuxR systems and the highly variable LuxR solo organization can be due to the adaptation of these bacteria to live in mixed communities and the ability to colonize several different environments. Unlike some bacterial species that harbor LuxI/R systems, which colonize specific niches upon reaching high cell densities, fluorescent pseudomonads may have increased their genetic plasticity to be part of mixed complex communities.

Based on sequence similarity, invariant amino acids of the IBD, and conservation of the flanking genes, we have placed LuxR solos into putative ortholog subgroups. The identification of a few LuxR solos which do not cluster into these subgroups having uncommon flanking genes and primary structure suggests that other LuxR solo subgroups exist. Nine different subgroups of LuxR solos have been mapped here, which included the well-studied subgroup of PAB LuxR solos and the other eight uncharacterized subgroups. Several previous studies have shown that PAB LuxR solos regulate the adjacently located *pip* gene in response to a plant compound. Members of this subfamily are characterized by few substitutions of two important amino acids in the autoinducer-binding site (34, 36, 55, 56). Our analysis revealed that PAB LuxR solos are very widespread among P. fluorescens sequenced genomes, especially among P. putida, probably due to its role in adapting to life next to the plants. Similarly, few members of the subgroup A, characterized by *luxR* solos flanked by two very conserved genes encoding a ferredoxin-NADP^+^ reductase and a 23S rRNA methyltransferase, were previously described, such as PpoR from P. putida (57). These studies revealed that PpoR plays an important role in iron acquisition; however, the molecular mechanism of the response of this subgroup of LuxR solos remains unknown. Subgroup A is the most widespread among P. fluorescens species and could be involved in both inter- and intraspecific processes relevant to the fitness of the P. fluorescens bacterial group, such as the control of some oxidation reactions associated with the rhizosphere, where the levels of toxic bioproducts of the aerobic metabolism of the plant are very high (58, 59). For the other subgroups of LuxR solos, there are no reports on their function and response/regulation. Interestingly, we observed a flexible rearrangement of the genomic context flanking different *luxR* solos and also a variable distribution and abundance of different subgroups among the species. It is possible that LuxR solos with different functions were acquired by these bacteria from different sources by horizontal gene transfer or genomic rearrangement events, as it is known to be highly prevalent in many *Pseudomonas* spp. (20, 60, 61). LuxR solos present in the same genome showed different levels of relatedness to each other, suggesting possible different origins and also possible different ligand binding properties.

To date, there are very few functionally characterized LuxR solos with known ligands (26, 33, 37–39). Our modeling analysis revealed that only two subgroups of LuxR solo are likely to bind and respond to AHL signals. One of these is subgroup A; however, our molecular and biochemical studies did not provide evidence for AHL binding. Alternatively, they may act independently of AHLs or may bind to different or modified AHL-like molecules produced by neighboring bacteria living in the same mixed community. As this subgroup is widespread among fluorescent *Pseudomonas* isolated from the rhizosphere, there could also be a possibility of sensing AHL-like molecules produced by the plant host. Prior studies have shown that AHL availability is higher in the rhizosphere than in the bulk soil (62); it is most likely that various concentrations or conditions of AHLs are needed for a response by this subgroup. Moreover, it cannot be excluded that some LuxR solos can act independently without the need of an inducing ligand, as previously reported (63). Alternatively, it is also a possibility that a ligand molecule is endogenously produced upon a stimulus, being an intracellular messenger. Additional studies are therefore required to understand the molecular mechanisms of these LuxR solo subfamilies. Non-AHL-binding LuxR solo subgroups could have evolved to respond to different signals, playing different roles in cell-cell communication, or having other more classic gene regulatory mechanisms. In particular, differences in the binding pocket conformation possibly suggest different inducer specificity and could result from the adaptation and evolutionary process to colonize, compete, and persist in different environments.

Our *in silico* analysis showed that several LuxR solos occur in a transcriptional unit with the neighboring genes; nevertheless, our expression analysis of promoter regions of flanking genes evidenced that, most often, their regulation is not under the nearby LuxR solo’s control. This suggests that LuxR solos could have evolved to have different target functions, that the expression studies performed here could be influenced by the absence of the LuxR solo ligands/signal molecules, or that the environmental growth conditions were not appropriate for LuxR solo function.

In summary, this study provides a large picture of LuxR solo distribution, classification, and abundance among the fluorescent pseudomonads group. The results highlight the existence of novel LuxR solos belonging to different subgroups that are likely to be involved in establishing possible novel communication networks or to have other regulatory responses. LuxR solos could have evolved away from QS systems (64) to respond to other endogenous or exogenous signals, expanding the regulatory networks for interspecies and interkingdom communication. Future work needs to establish their role and the signals they respond to in the plant-associated microbiome.

## MATERIALS AND METHODS

### Bacterial species, culture conditions, and genome sequencing.

The bacterial strains used in this work were as follows: P. putida 16A and *P. oleovorans* AG1003 (isolated from rhizosphere and endosphere rice plants collected during this project), P. fluorescens F113 (65) and *P. jessenii* DSM 17150 (Leibniz Institute DSMZ-German Collection of Microorganisms and Cell Cultures GmbH, Germany). All strains were grown in liquid Luria-Bertani (LB), King’s broth (KB), or M9 medium at 30°C under moderate shaking (120 rpm). When required, antibiotics for *Pseudomonas* strain growth were added at the following concentrations: nitrofurantoin (Nf), 100 μg ml^−1^; ampicillin (Amp), 100 μg ml^−1^. The mutants of each strain (carrying a knockout mutation of the *luxR* solo gene) were grown using 100 μg ml^−1^ kanamycin (Km) as antibiotic. E. coli DH5α, S17, and BL21(DE3) were routinely grown at 37°C in LB broth, and antibiotics were added when required at the following concentrations: Amp, 100 μg ml^−1^; tetracycline, 15 μg ml^−1^. AHLs were obtained from Sigma-Aldrich (St. Louis, MO, USA).

The complete genomes of 20 fluorescent *Pseudomonas* spp. were sequenced with the Illumina MiSeq platform using 150-bp paired-end reads and according to the tagmentation Illumina Nextera XT protocol (Illumina Inc., San Diego, CA, USA). The sequencing was performed by the Exeter University (UK). Sequenced genomic DNA was assembled using Spades 3.9.03 (66), and the assembled sequence was annotated using the NCBI Prokaryotic Genome Annotation Pipeline (PGAP). Genomes were also annotated using RAST (Rapid Annotation using Subsystem Technology) server (67), uploaded to the Integrated Microbial Genomes and Metagenomes (IMG/M) database, and automatically annotated using annotation pipeline IMG Annotation Pipeline v.4.16.6 (68).

### Plasmid and recombinant DNA techniques.

The plasmids, constructs, and set of specific primers (Sigma-Aldrich) used in this study are listed in Table 1. pGEM-T Easy vector (Promega Corp., Madison, WI, USA) was used for cloning. When necessary, 5-bromo-4-chloro-3-indolyl-β-d-galactoside (X-Gal) was added at a final concentration of 80 μg ml^−1^. Routine DNA manipulation steps, such as digestion with restriction enzymes, agarose gel electrophoresis, purification of DNA fragments, ligation with T4 DNA ligase, and transformation of E. coli, were performed as described previously (69). Plasmids were purified by using EuroGold columns (EuroClone, Milan, Italy); total DNA was isolated by Sarkosyl-pronase lysis, as described previously (70). Digestion with restriction enzymes was conducted according to the supplier’s instructions (New England BioLabs, USA). DNA was ligated with T4 DNA ligase (New England BioLabs, USA) according to the manufacturer’s recommendations.

**TABLE 1:** Plasmids and primers used

Plasmid or primer	Relevant features or sequence	Reference or source
Plasmids		
pGEM-T	Cloning vector; Amp^r^	Promega
pMP220	Promoter probe vector; IncP; Tc^r^	92
pBBR1MCS-5	Broad-host-range vector; Gm^r^	72
pLAFR3	Broad-host-range vector; IncP; Tc^r^	93
pEX19Gm	Suicide vector for making deletion mutants, Gm^r^	94
pETM-11	His_6_-tagged protein expression vector	Addgene, Watertown, MA
pUC4K	pUC7 derivative, Amp^r^ and Km^r^	Addgene, Watertown, MA
pEX19-PpuR16R	PpuR16R sequence depleted of 20 bp, cloned in pEX19Gm	This study
pEX19-PpuR16R_2	PpuR16R_2 sequence depleted of 20 bp, cloned in pEX19Gm	This study
pEX19-PfluR_113	PfluR_113 sequence depleted of 20 bp, cloned in pEX19Gm	This study
pEX19-PjeR	PjeR sequence depleted of 20 bp, cloned in pEX19Gm	This study
pEX19-PolR	PolR sequence depleted of 20 bp, cloned in pEX19Gm	This study
pPppu16R220	Ppu16R promoter cloned in pMP220	This study
pPferr220	Ferredoxin NADP reductase promoter cloned in pMP220	This study
pP23S220	23S rRNA methyltransferase promoter cloned in pMP220	This study
pPppu16R2_220	Ppu16R_2 promoter cloned in pMP220	This study
pPfluR220	PfluR_113 promoter cloned in pMP220	This study
pPmoaF220	MoaF promoter cloned in pMP220	This study
pPjeR220	PjeR promoter cloned in pMP220	This study
pPsperm220	Spermidine permease promoter cloned in pMP220	This study
pPolR220	PolR promoter cloned in pMP220	This study
pPputr220	Putrescine importer promoter cloned in pMP220	This study
pBBR-PfluR	PfluR_113 cloned in ΔPfluR_113	This study
pETM-Ppu16R	Ppu16R sequence cloned in pETM-11	This study
Primers		
KmR1	CAACTCTGGCGCATCGGGCT	This study
KmR2	GCGTAATGCTCTGCCACACA	This study
P16A_SOLO_EXT	GAGATTTCCTACACTTCGTTC	This study
P16A_SOLO2_EXT	AGATCGTCAACGACGGC	This study
PF113_SOLO_EXT	TGGTCAGCGAGAGTTTCGTC	This study
PJES_SOLO_EXT	GTGCTCGCTAAAGGATTCAG	This study
POLEOV_SOLO_EXT	ACTCTAGGCCAGGGTGGG	This study
FW_F113_SOLO_compl_Xba	TCTAGACTGTGGGAAGTGGTCA	This study
RV_F113_SOLO_compl_Kpn	GGTACCTGGTTGATCAGAGGAA	This study

### Genomic mutant construction and their complementation.

In-frame deletions of the *luxR* solo genes were generated using the pEX19Gm plasmid as described previously (71). Briefly, each *luxR* solo gene sequence, synthetized by Twist Bioscience company (South San Francisco, CA), is listed in Table S1 in the supplemental material. The design of the constructs was performed as follows: internal fragments of 20 bp from each gene of interest were deleted and replaced with a restriction site (BamHI or SmaI) in order to clone inside the Km gene cassette previously extracted from pUC4K. Sequentially, the fragments were excised with Kpn and XbaI restriction enzymes and cloned in the corresponding site in pEX19Gm. The resulting pEX19Gm-derivative plasmids, listed in Table 1, were introduced by biparental conjugation in the corresponding *Pseudomonas* genomes. Clones with a chromosomal insertion of the pEX19Gm plasmids were selected on LB agar plates supplemented with 40 μg ml^−1^ gentamicin (Gm) and 100 μg ml^−1^ Nf. Plasmid excision from the chromosome was subsequently selected on LB agar plates supplemented with 10% (wt/vol) sucrose. All the mutants were verified by PCR using primers (Table 1) specific to the Km cassette and to the genomic DNA sequences upstream and downstream from the targeted genes.

For complementation analysis, the encoding regions of each *luxR* solo full-length genes were amplified by the primers listed in Table 1. The PCR products were digested with restriction enzymes and then cloned in the expression vector pBBR1MCS-5 (72) digested with the same enzymes. The complementation constructs were introduced into corresponding mutants by biparental mating selected for Km^r^ and Gm^r^ and confirmed by PCR analysis.

### β-Galactosidase activity assay.

To identify possible target genes, the promoter regions of several genes adjacent to each *luxR* solo studied were synthetized by Twist Bioscience company (South San Francisco, CA) and cloned into promoter probe vector pMP220, which harbors a promoterless *lacZ* gene, as described in Tables 1 and S1. pMP derivative constructs were then introduced independently into the wild-type strain and each corresponding *luxR* solo mutant by conjugation. β-Galactosidase assays were performed as previously described by Miller and Lee (73), with the modifications of Stachel et al. (74). Average Miller unit values and standard deviations were calculated from three independent experiments. When necessary, AHLs (C_4_ homoserine lactone [HSL], C_6_-HSL, OHC_6_-HSL, OC_6_-HSL, C_8_-HSL, OHC_8_-HSL, OC_8_-HSL, C_10_-HSL, OHC_10_-HSL, OC_10_-HSL, C_12_-HSL, OHC_12_-HSL, and OC_12_-HSL) were added at the final concentration of 1 μM as well as a cocktail of polyamines (putrescine, spermine, and spermidine) (Sigma-Aldrich, St. Louis, MO, USA) at a final concentration of 0.1 mM.

### Statistical analysis.

For analysis of statistical significance, the data were analyzed using GraphPad Prism’s *t* test or analysis of variance (ANOVA), and a *P* value of <0.05 was considered significant for all experiments.

### Fluorescent pseudomonad strain isolation.

A set of 20 fluorescent pseudomonad strains were purified from a laboratory collection of rhizospheres and endospheres of rice plants (75), stored in glycerol at −80°C. The samples were plated on KB agar medium supplemented with an iron chelator such as ethylendiamine-*N*,*N*′-diacetic acid (EDDA) (Sigma-Aldrich, St. Louis, MO, USA). Fluorescent pseudomonad strains producing fluorescent siderophores under iron-limited conditions were detected, exposing the plates under UV rays. The fluorescent colonies were isolated and stored at −80°C in a 18% glycerol suspension.

### Protein and sequence data download.

Protein FASTA sequences of 601 genomes from 17 *Pseudomonas* species were downloaded from PATRIC database (44).

### Detection of LuxR/*luxR* and LuxI/*luxI* proteins/genes.

Hidden Markov model (HMM) recognizers were collected from PFAM for the autoinducer-binding domain and the GerE domain typical of luxR and autoinducer synthase domain from InterPro for identification of LuxI proteins. These HMM recognizers were used to identify LuxR and LuxI proteins among all *Pseudomonas* strains using hmmsearch tool (76). Hits with an E value less than 10^−10^ were taken as potential homologues of QS genes.

### Phylogenetic analysis.

Phylogenetic trees for all the *Pseudomonas* strains were built using the MEGAX program package (77) installed from http://www.megasoftware.net using the neighbor-joining method and then visualized using ggtree package in R (78).

### Homology modeling and structural alignments.

Five web-based servers were exploited to build the three-dimensional (3D) homology models of the IBD of each LuxR solo studied. The top-score models generated by the different approaches were then ranked and validated by the protein model quality predictor ProQ (79), including PSIPRED (80) for secondary structure prediction.

The top-scored model of the prototype of subgroup A, Ppu16R (having the predicted LGscores and MaxSub values of 4.155 and 0.336, respectively), was obtained by M4T (81), based on two templates: SdiA from E. coli (PDB identifier [ID] 4Y13) (50) and CviR from Chromobacterium violaceum (PDB ID 3QP6) (82).

The top-scored model of the prototype of subgroup B (having the predicted LGscores and MaxSub values of 4.078 and 0.725, respectively) was obtained by M4T (81), based on two templates: QscR from P. aeruginosa (PDB ID 3SZT) (13) and SdiA from E. coli (PDB ID 4Y13) (50).

The top-scored model of the prototype of subgroup D, PfluR_113 (having the predicted LGscores and MaxSub values of 4.078 and 0.725, respectively), was obtained by RaptorX (83), based on CviR from C. violaceum (PDB ID 3QP5) (82) as a template.

Regarding the subgroup E, two members, PolR and PjeR, have been modeled. In detail, the top-scored model of PolR (having the predicted LGscores and MaxSub values of 4.063 and 0.870, respectively) was obtained by Phyre2 (84), based on CviR from C. violaceum (PDB ID 3QP5) (82) as a template. The top-scored model of PjeR (having the predicted LGscores and MaxSub values of 4.205 and 0.580, respectively) was obtained by M4T (81), based on five templates: TraR from Sinorhizobium fredii (PDB ID 2Q0O) (85) and from Agrobacterium tumefaciens (PDB ID 1H0M) (86), SdiA from E. coli (PDB ID 4LFU) (87), CviR from C. violaceum (PDB ID 3QP5) (82), and QscR from P. aeruginosa (PDB ID 3SZT) (13).

The top-scored model of the prototype of subgroup F (having the predicted LGscores and MaxSub values of 4.052 and 0.931, respectively) was obtained by RaptorX (83), based on CviR from C. violaceum (PDB ID 3QP5) (82) as a template.

The top-scored model of the prototype of subgroup G (having the predicted LGscores and MaxSub values of 4.062 and 0.811, respectively) was obtained by M4T (81), based on CviR from C. violaceum (PDB ID 3QP5 and 3QP6) (82) as the template.

The top-scored model of the prototype of subgroup H, Ppu16R_2 (having the predicted LGscores and MaxSub values of 4.556 and 0.560, respectively), was obtained by RaptorX (83), based on TraR from *S. fredii* (PDB ID 2Q0O) (85) as a template.

The top-scored model of the prototype of subgroup I (having the predicted LGscores and MaxSub values of 4.037 and 0.737, respectively) was obtained by Phyre2 (84), based on SdiA from E. coli (PDB ID 4LFU) (87) as a template.

Sequence alignment was performed by Expresso (88), exploiting structural aligners algorithms such as SAP (89) or TMalign (90). Each subgroup prototype was also aligned with all the canonical QS LuxR proteins, whose X-ray structures are available: TraR from A. tumefaciens (PDB ID 1H0M [86]) and from *S. fredii* NGR234 (PDB ID 2Q0O [85]), LasR (PDB ID 3IX3 [91]) and QscR (PDB ID 3SZT [13]) from P. aeruginosa, CviR from C. violaceum (PDB ID 3QP1 [82]), and SdiA from E. coli (PDB ID 4Y13 [50]). The structure-based homology model of OryR from *X. oryzae* (49), the prototype of subgroup C, was also included in the structural-based multiple alignment.

### Data availability.

Each whole-genome shotgun project has been deposited at DDBJ/ENA/GenBank and is accessible at BioProject under accession ID PRJNA701950. The whole-genome shotgun project of *Pseudomonas* sp. 18_A has been deposited under the accession JAFGYG000000000, and the version described in this paper is version JAFGYG010000000. The whole-genome shotgun project of *Pseudomonas* sp. 29_B has been deposited under the accession JAFGYH000000000, and the version described in this paper is version JAFGYH010000000. The whole-genome shotgun project of *Pseudomonas* sp. 32_A has been deposited under the accession JAFGYI000000000, and the version described in this paper is version JAFGYI010000000. The whole-genome shotgun project of *Pseudomonas* sp. 43(2021) has been deposited under the accession JAFGYJ000000000, and the version described in this paper is version JAFGYJ010000000. The whole-genome shotgun project of *Pseudomonas* sp. 21_B has been deposited under the accession JAFGYK000000000, and the version described in this paper is version JAFGYK010000000. The whole-genome shotgun project of *Pseudomonas* sp. 67(2021) has been deposited under the accession JAFGYL000000000, and the version described in this paper is version JAFGYL010000000. The whole-genome shotgun project of *Pseudomonas* sp. 69_B has been deposited under the accession JAFGYM000000000, and the version described in this paper is version JAFGYM010000000. The whole-genome shotgun project of *Pseudomonas* sp. 71_D has been deposited under the accession JAFGYN000000000, and the version described in this paper is version JAFGYN010000000. The whole-genome shotgun project of *Pseudomonas* sp. 74_A has been deposited under the accession JAFGYO000000000, and the version described in this paper is version JAFGYO010000000. The whole-genome shotgun project of *Pseudomonas* sp. 78_B has been deposited under the accession JAFGYP000000000, and the version described in this paper is version JAFGYP010000000. The whole-genome shotgun project of *Pseudomonas* sp. 79_C has been deposited under the accession JAFGYQ000000000, and the version described in this paper is version JAFGYQ010000000. The whole-genome shotgun project of *Pseudomonas* sp. 81_B has been deposited under the accession JAFGYR000000000, and the version described in this paper is version JAFGYR010000000. The whole-genome shotgun project of *Pseudomonas* sp. 86_A has been deposited under the accession JAFGYS000000000, and the version described in this paper is version JAFGYS010000000. The whole-genome shotgun project of *Pseudomonas* sp. 95_A has been deposited under the accession JAFGYT000000000, and the version described in this paper is version JAFGYT010000000. The whole-genome shotgun project of *Pseudomonas* sp. 100_A has been deposited under the accession JAFGYU000000000, and the version described in this paper is version JAFGYU010000000. The whole-genome shotgun project of *Pseudomonas* sp. 16_A has been deposited under the accession JAFGYV000000000, and the version described in this paper is version JAFGYV010000000. The whole-genome shotgun project of *Pseudomonas* sp. 51_B has been deposited under the accession JAFGYW000000000 , and the version described in this paper is version JAFGYW010000000. The whole-genome shotgun project of *Pseudomonas* sp. 50_B has been deposited under the accession JAFGYX000000000, and the version described in this paper is version JAFGYX010000000. The whole-genome shotgun project of *Pseudomonas* sp. 30_B has been deposited under the accession JAFGYY000000000, and the version described in this paper is version JAFGYY010000000. The whole-genome shotgun project of *Pseudomonas* sp. 58(2021) has been deposited under the accession JAFGYZ000000000, and the version described in this paper is version JAFGYZ010000000.
