# Erratum for Bez et al., “LuxR Solos from Environmental Fluorescent Pseudomonads”

**DOI:** 10.1128/mSphere.00853-21

**Published:** 2021-10-27

**Authors:** Cristina Bez, Sonia Covaceuszach, Iris Bertani, Kumari Sonal Choudhary, Vittorio Venturi

**Affiliations:** a International Centre for Genetic Engineering and Biotechnology, Trieste, Italy; b Institute of Crystallography, CNR, Trieste, Italy; c University of California San Diego, La Jolla, California, USA

## ERRATUM

Volume 6, no. 2, e01322-20, 2021, https://doi.org/10.1128/mSphere.01322-20. This article was published on 31 March 2021 with the 2nd author’s surname misspelled. The byline was updated in the current version, posted on 27 October 2021.

