# Supplementary material for: LuxR Solos from Environmental Fluorescent Pseudomonads
Source: mSphere. 2021 Mar 31;6(2):e01322-20. doi: 10.1128/mSphere.01322-20 (PMC8546723; doi:10.1128/mSphere.01322-20)
Supplement: FIG S1 [file msphere.01322-20-sf001.pdf]

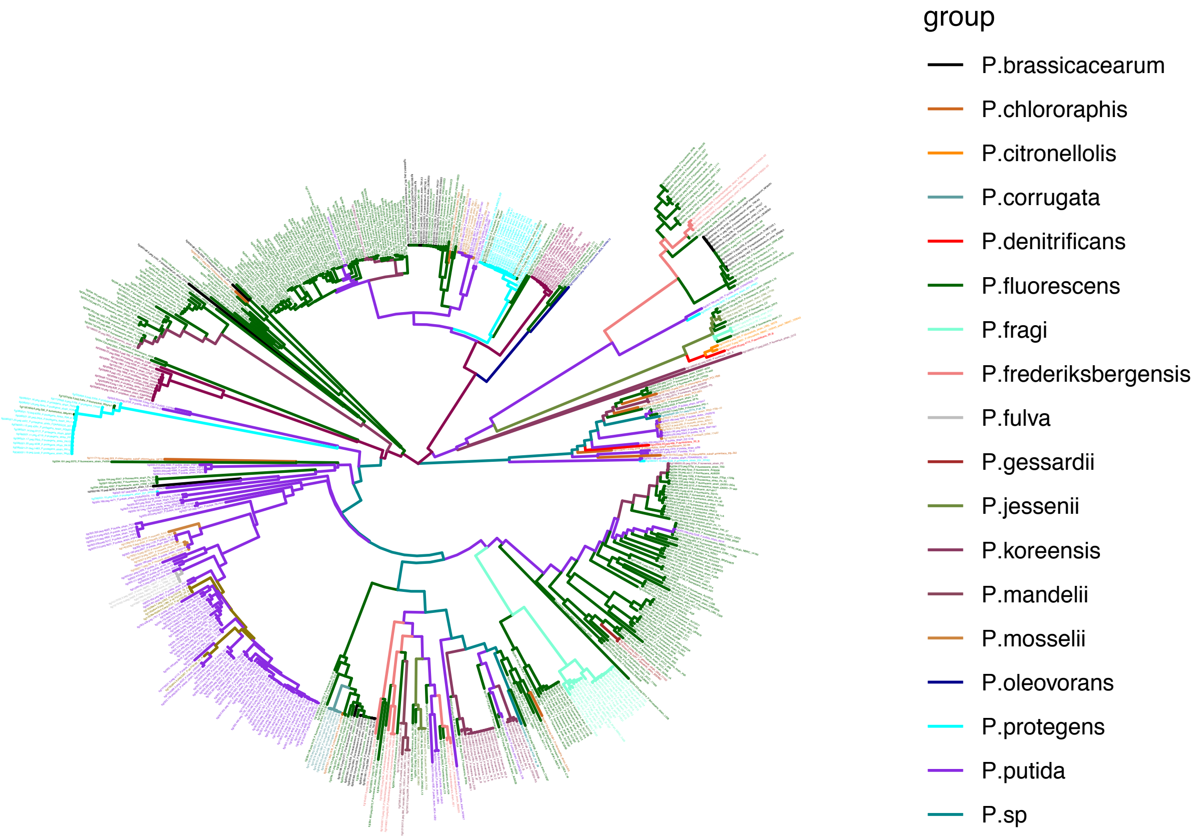
group
P.brassicacearum
P.chlororaphis
P.citronellolis
P.corrugata
P.denitrificans
P.fluorescens
P.fragi
P.frederiksbergensis
P.fulva
P.gessardii
P.jessenii
P.koreensis
P.mandelii
P.mosselii
P.oleovorans
P.protegens
P.putida
P.sp
P.sp.FGI182
P.viridiflava
