# Supplementary figures and images for: LuxR Solos from Environmental Fluorescent Pseudomonads
Source: mSphere. 2021 Mar 31;6(2):e01322-20. doi: 10.1128/mSphere.01322-20 (PMC8546723; doi:10.1128/mSphere.01322-20)

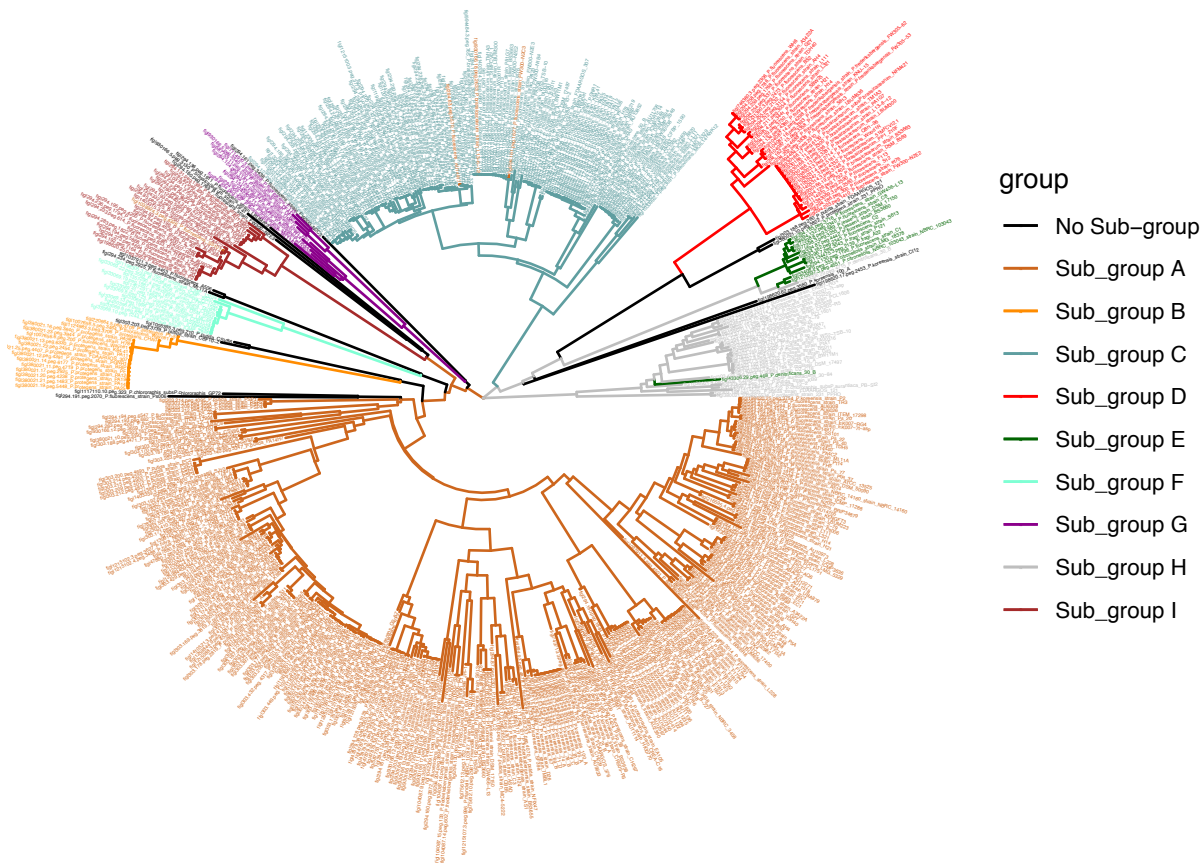

Supplement: FIG S2 [file msphere.01322-20-sf002.pdf]

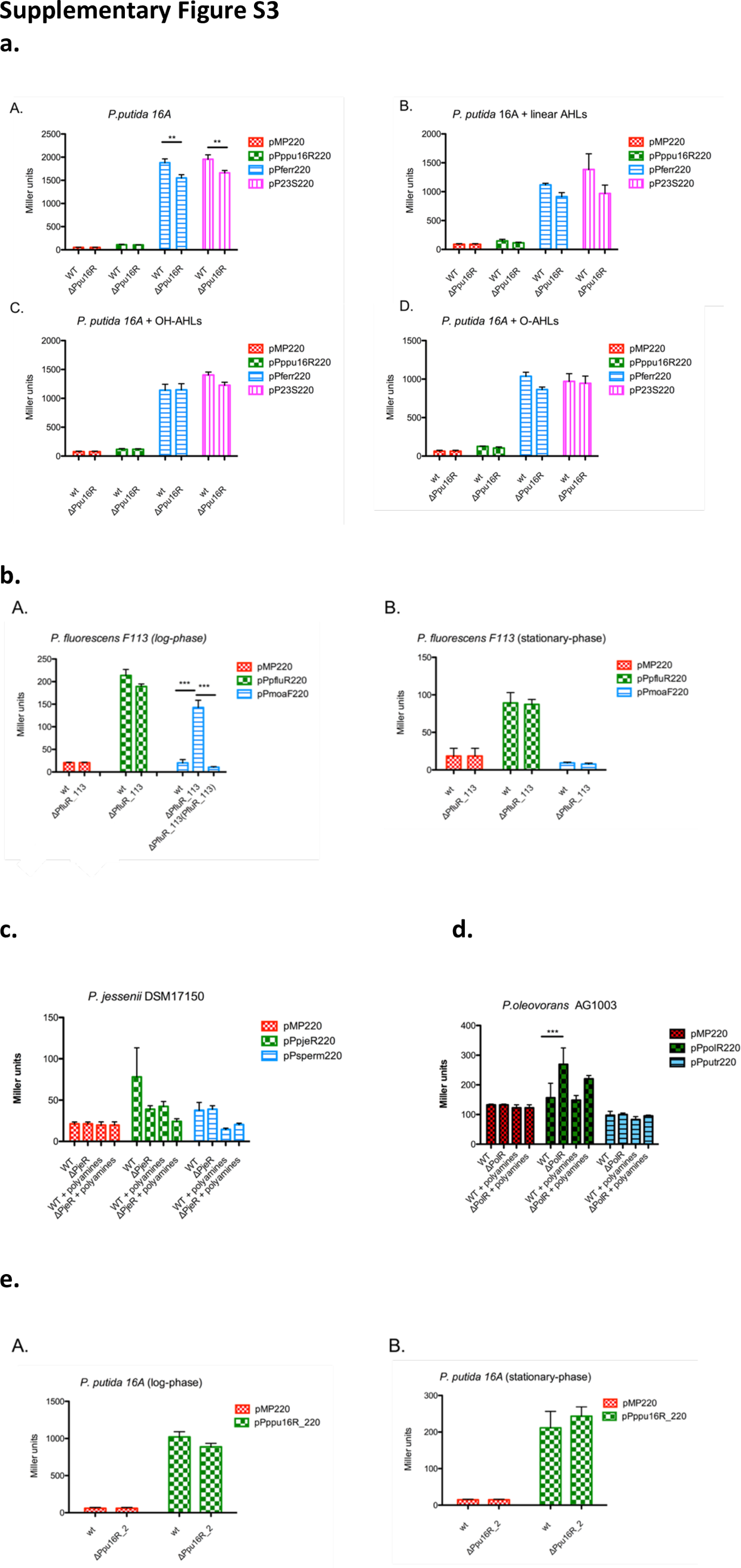

Supplement: FIG S3 [file msphere.01322-20-sf003.tif]

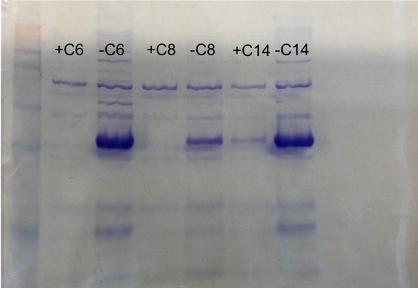

Supplement: FIG S4 [file msphere.01322-20-sf004.tif]
