# Supplementary material for: LuxR Solos from Environmental Fluorescent Pseudomonads
Source: mSphere. 2021 Mar 31;6(2):e01322-20. doi: 10.1128/mSphere.01322-20 (PMC8546723; doi:10.1128/mSphere.01322-20)
Supplement: TABLE S4 [file msphere.01322-20-st004.pdf]

		1	2	3	4	5	6	7	8	9
Sub_groupA	1	100.00	25.88	22.52	21.21	23.31	17.83	13.79	13.69	15.05
Sub_groupG	2	25.88	100.00	16.85	17.31	21.92	18.21	11.30	13.79	14.64
Sub_groupI	3	22.52	16.85	100.00	18.63	18.52	15.57	14.24	10.22	14.49
Sub_groupH	4	21.21	17.31	18.63	100.00	16.60	17.38	10.10	11.41	12.95
Sub_groupF	5	23.31	21.92	18.52	16.60	100.00	13.98	12.67	14.51	15.19
Sub_groupC	6	17.83	18.21	15.57	17.38	13.98	100.00	17.43	15.49	12.15
Sub_groupD	7	13.79	11.30	14.24	10.10	12.67	17.43	100.00	15.11	14.74
Sub_groupE	8	13.69	13.79	10.22	11.41	14.51	15.49	15.11	100.00	9.47
Sub_groupB	9	15.05	14.64	14.49	12.95	15.19	12.15	14.74	9.47	100.00
